# Evaluation of LiF:Mg,Ti (TLD-100) for Intraoperative Electron Radiation Therapy Quality Assurance

**DOI:** 10.1371/journal.pone.0139287

**Published:** 2015-10-01

**Authors:** Raffaele Liuzzi, Federica Savino, Vittoria D’Avino, Mariagabriella Pugliese, Laura Cella

**Affiliations:** 1 Institute of Biostructure and Bioimaging, National Research Council (CNR), Naples, Italy; 2 Department of Physics, Federico II University, Naples, Italy; Technische Universitaet Muenchen, GERMANY

## Abstract

**Background:**

Purpose of the present work was to investigate thermoluminescent dosimeters (TLDs) response to intraoperative electron radiation therapy (IOERT) beams. In an IOERT treatment, a large single radiation dose is delivered with a high dose-per-pulse electron beam (2–12 cGy/pulse) during surgery. To verify and to record the delivered dose, *in vivo* dosimetry is a mandatory procedure for quality assurance. The TLDs feature many advantages such as a small detector size and close tissue equivalence that make them attractive for IOERT as *in vivo* dosimeters.

**Methods:**

LiF:Mg,Ti dosimeters (TLD-100) were irradiated with different IOERT electron beam energies (5, 7 and 9 MeV) and with a 6 MV conventional photon beam. For each energy, the TLDs were irradiated in the dose range of 0–10 Gy in step of 2Gy. Regression analysis was performed to establish the response variation of thermoluminescent signals with dose and energy.

**Results:**

The TLD-100 dose-response curves were obtained. In the dose range of 0–10 Gy, the calibration curve was confirmed to be linear for the conventional photon beam. In the same dose region, the quadratic model performs better than the linear model when high dose-per-pulse electron beams were used (F test; p<0.05).

**Conclusions:**

This study demonstrates that the TLD dose response, for doses ≤10Gy, has a parabolic behavior in high dose-per-pulse electron beams. TLD-100 can be useful detectors for IOERT patient dosimetry if a proper calibration is provided.

## Introduction

Intraoperative electron radiation therapy (IOERT) is a treatment modality where a large single radiation dose (from 8 to 30 Gy) is delivered during surgery, either with or without resection of a neoplastic mass. In recent years, IOERT has been used in early stage cancer as an exclusive radiation modality (23–30 Gy) [1–4], or as a boost (8–20 Gy), especially for breast tumours [5–10]. Accelerators specially designed for IEORT and unshielded operating theaters have been manufactured promoting a widespread use of this radiotherapy technique. These mobile electron accelerators are characterized by output rates (about 2–12 cGy/pulse) several times higher than conventional accelerators (0.01–0.06 cGy/pulse) [11]. The high dose-per-pulse reduces the irradiation time during the surgery (typically 10 Gy are delivered in less than 1 minute). Because of the very high planned dose in an IOERT treatment, *in vivo* dosimetry is highly recommended in order to: verify the correct delivery of irradiation; apply possible monitor units corrections and record the dose received by patients [12]. Dosimeters like MOSFETs and radiochromic films have been routinely used for this purpose [13–15]. Although thermoluminescent dosimeters (TLDs) are well-established for *in vivo* dose verification, their response to IOERT beams has not been yet investigated. TLDs are widely used to determine patient doses in radiation diagnostics and external beam radiotherapy. The dose ranges of interest are approximately 0.1–100 mGy for clinical X-ray diagnostics and 1–5 Gy for radiotherapy [16, 17]. TLDs possess advantageous characteristics that allow for easy use and reproducibility in both clinical and research settings, such as their small size, reusability and few correction factors to be applied [18, 19]. The lithium fluoride doped with magnesium and titanium LiF:Mg,Ti (TLD-100) is the most widely used TLD in routine personal dosimetry, environmental monitoring, space dosimetry and clinical dosimetry. Furthermore, recently the use of TLDs has been proposed for radiopharmaceutical nanoagents [20].

This popularity is due to its approximate tissue equivalence (effective atomic number of 8.2, similar to 7.4 for tissue); low signal fading (5–10% per year), wide linear response range (10μGy- 10Gy); and high sensitivity for very low dose measurements [21, 22]. Due to their small size, TLDs are convenient for point dose measurements in phantoms as well as for *in vivo* dosimetry on patients during radiotherapy treatment [23].

The thermoluminescent response of TLD-100 is not linear for large doses. At higher doses, above 10 Gy, it exhibits a supralinear behavior [24]; this supralinearity has been one of the main drawbacks to their use in high-dose dosimetry. For this reason, the precise knowledge of the onset of the supralinear response is of practical importance [25]. Also, the TLD response is beam energy and modality dependent [26]. To the best of our knowledge, the response of TLDs irradiated by high dose-per-pulse electron beams has not been investigated yet.

In this study, we have investigated the feasibility of TLDs as *in vivo* dosimeters in IOERT treatments in the dose range of 0–10 Gy (inherent to tumor boost). Their use was conceived for independent verification of treatment dose delivery in order to improve patient quality assurance. Experimental dose-response data were obtained exposing LiF:Mg,Ti dosimeters to three different electron beams (energy 5, 7 and 9 MeV) produced by an IOERT specific accelerator. In addition, the dose-response curve for conventional 6MV photon beam was determined. The functional form of the different dose-response curves was determined.

## Materials and Methods

The irradiation experiments were performed using 5, 7 and 9 MeV electron beams. An additional reference irradiation experiment was carried out by a 6 MV photon beam.

The electron beams were produced by a mobile linear accelerator Novac7 (Hitesys, Italy) specifically designed for IOERT and installed at the University of Naples Federico II, Italy. The Novac7 produces high dose-per-pulse electron beams with a duration of 4 μs and pulse repetition rate of 5 Hz. The nominal energies are 3, 5, 7, and 9 MeV. The accelerator is equipped with a 3D movable arm that can be placed on the operating field while the beam is collimated by Perspex cylindrical applicators having 4, 5, 6, 8, and 10 cm inner diameters. The applicators length is 80 cm, excluding the 10 cm applicator which is 100 cm long. Dose-per-pulse rates in the range of 2–8 cGy/pulse can be obtained by varying the applicator’s diameter and energy [11].

The photon beam was produced by a Primus (Siemens, Germany) also installed at the University “Federico II”. This accelerator can deliver photon beams with nominal energy of 6MV, operate with a dose rate of 2 Gy/minute for a field size of 10x10 cm^2^ and a dose-per-pulse rate in the range of 0.015 cGy/pulse. The system of collimation of the beam is constituted by a pair of conventional tungsten collimators and by a multileaf collimator.

The conventional 6 MV photon beam irradiation has been performed to obtaining a TLD dose-response curve to be used as a benchmark for the applied method of analysis.

### Dosimeters and annealing schedule

The TLDs used in this work were the LiF:Mg,Ti (TLD-100) (Harshaw Chemical Company) chips with nominal dimensions of 3.2 *×* 3.2 *×* 0.89 mm^3^, lower dose limit of 10 pGy and spatial resolution of 2 mm [27, 28]. Prior to each irradiation, TLDs were annealed in air at 400°C for 1 hour, followed by a 2 hours annealing at 100°C and by rapid cooling to room temperature [8]. The readout of TLDs was performed by a Harshaw model 3500 manual TLD reader installed at the Department of Physics of the University of Naples Federico II. TLDs have been read at 300°C using a heating rate of 10°C/s to optimize the TL signal-to-background ratio in the high-temperature region. A continuous nitrogen flow was used to reduce chemiluminescence and spurious signals not related to the irradiation [24]. A total of 54 TLDs were used.

### Individual Calibration of the dosimeters

TLDs were calibrated in terms of absorbed dose to water using the 6 MV photon beam. TLDs were individually identified by a code and irradiated in the same geometrical conditions to obtain the individual sensitivity correction factor defined as:

$$S_{i}\;=\frac{R_{i}}{\bar{R}}$$

where *R*_*i*_ is the thermoluminescent response of each TLD and $\bar{R}$ is the mean of the responses of all TLDs. The *S*_*i*_ expresses the response variation of each individual dosimeter around the mean. Any dosimeter with a relative sensitivity value greater than ±10% of the mean value must be rejected [29].

**Fig 1:**
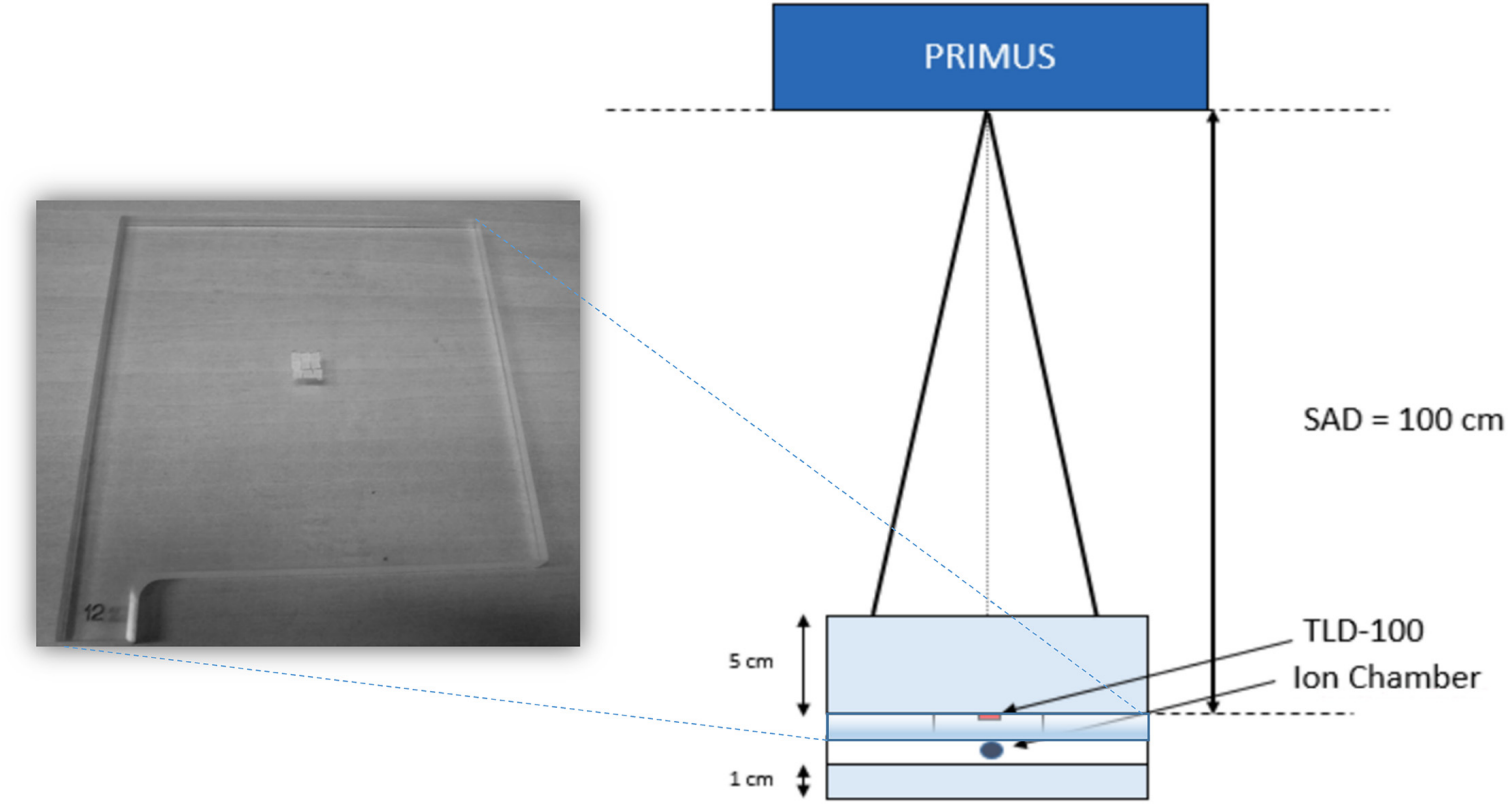
Experimental setup used to irradiate the Thermoluminescent Dosimeters (TLDs) with the 6 MV photon beam from the clinic linear accelerator Primus Siemens. SAD: Source Axis Distance.

TLDs were placed at a distance of 100 cm from the source (source-to-axis distance technique), at a depth of 5 cm in a water-equivalent phantom (RW3 slab phantom) [30] and irradiated with a beam size of 10x10 cm^2^ and a total dose of 2 Gy (Fig 1). Before the irradiation, the beam output (Gy/UM) was verified using a Farmer chamber type (PTW type 30001, Freiburg, Germany) according to the recommendations of the international protocol reported in [31]. During any TLD irradiation measurement, the delivered dose was separately measured by a ionization chamber to check the accuracy of the delivered dose (Fig 1).

### Irradiation of the TLD—experimental setup

Groups of 9 TLDs were housed in a cavity on purpose shaped in a slab of plexiglass and inserted in the water-equivalent phantom (Fig 1). Each group was irradiated with a single dose value ranging between 2 and 10 Gy in steps of 2Gy. In each measurement session, a group of 9 TLDs was not irradiated to obtain the background signal.

As a first step, the TLDs were irradiated with 6 MV photon beam. The irradiation was performed with the same experimental setup and procedure described for TLDs calibration.

**Fig 2:**
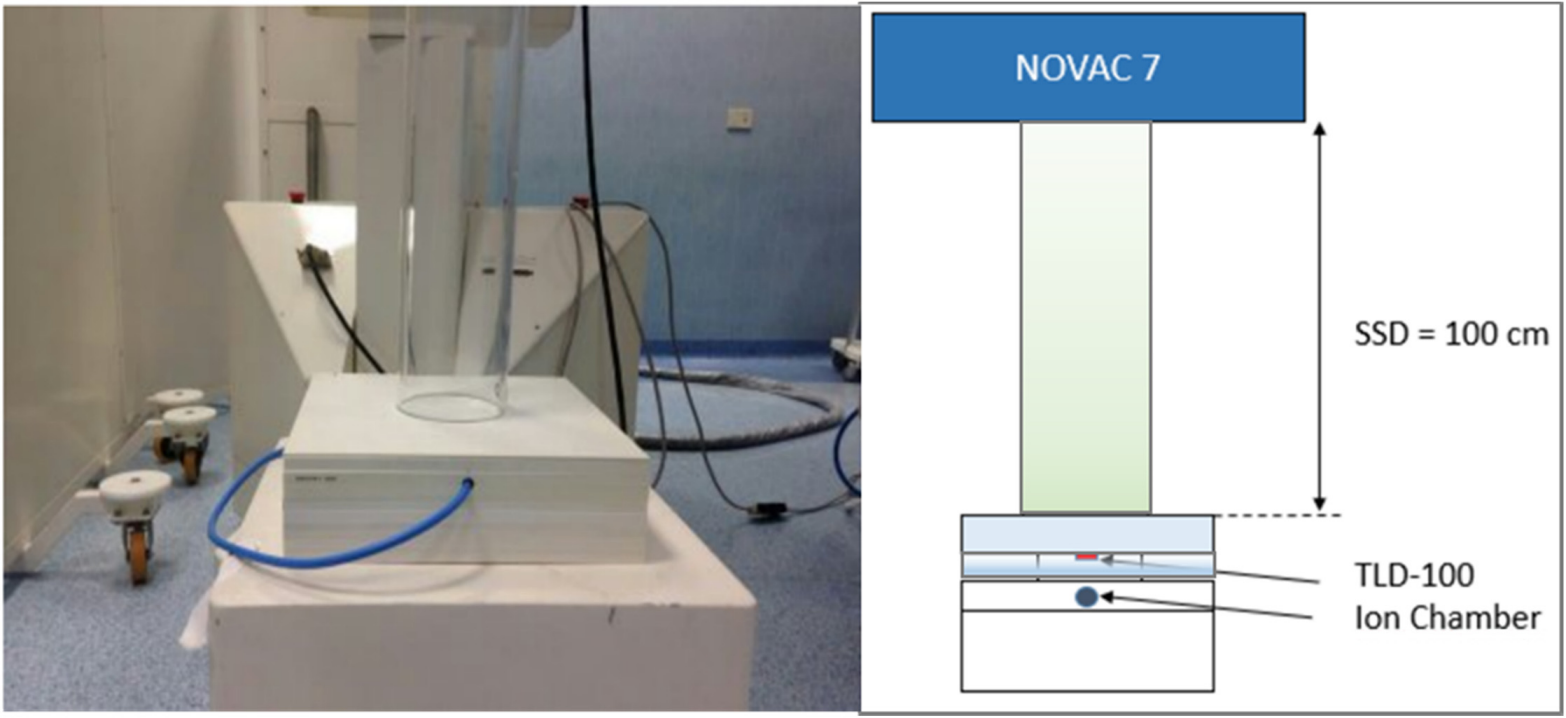
Experimental setup used to irradiate the Thermoluminescent Dosimeters (TLDs) with the 5, 7 and 9 MeV electron beams from the intra-operative linear accelerator Novac7. SSD: Source Surface Distance.

Next, the TLDs were irradiated with the electron beams (5-7-9 MeV) produced by the Novac7 at the depth of maximum dose of each electron energy (Table 1). The distance between the source and the upper surface of the phantom was of 100 cm (source surface distance technique), and the field diameter was of 10 cm (Fig 2). Each beam operates at different dose-rate (7.8, 8.1 and 9.3 GY/min for 5, 7 and 9 MeV energy beam). All beams characteristics are described in Table 1. Before each set of irradiation, the beam output (Gy/UM) was verified using an Advanced Markus chamber type (PTW type 34045, Freiburg, Germany) according to the protocol previously described in [11]. During any TLD irradiation measurement, the delivered dose was separately measured by a ionization chamber to check the accuracy of the delivered dose (Fig 2).

**Table 1:** Intraoperative mobile linear accelerator Novac 7: electron beams characteristics.

Energy (MeV)	Applicator (mm)	Dose-per-pulse (cGy/imp)	Dose-rate (Gy/min)	Dmax (mm)
5	100	2.6	7.8	8
7	100	2.7	8.1	11
9	100	3.1	9.3	13

### Statistical analysis

Statistical analysis was performed with MedCalc (MedCalc Software bvba, Ostend, Belgium) and OriginLab (OriginLab Corporation, Northampton, Massachusetts). For each dose-energy measurements, the mean response and standard error for the TLD’s group were calculated.

For all beams, regression analysis was performed on TLD response as a function of delivered doses. The goodness of the fit was evaluated by the R^2^ coefficient. To identify the model that best fits the experimental data the F-test was applied [32]. A *p* value less than 0.05 was considered statistically significant.

## Results

**Fig 3:**
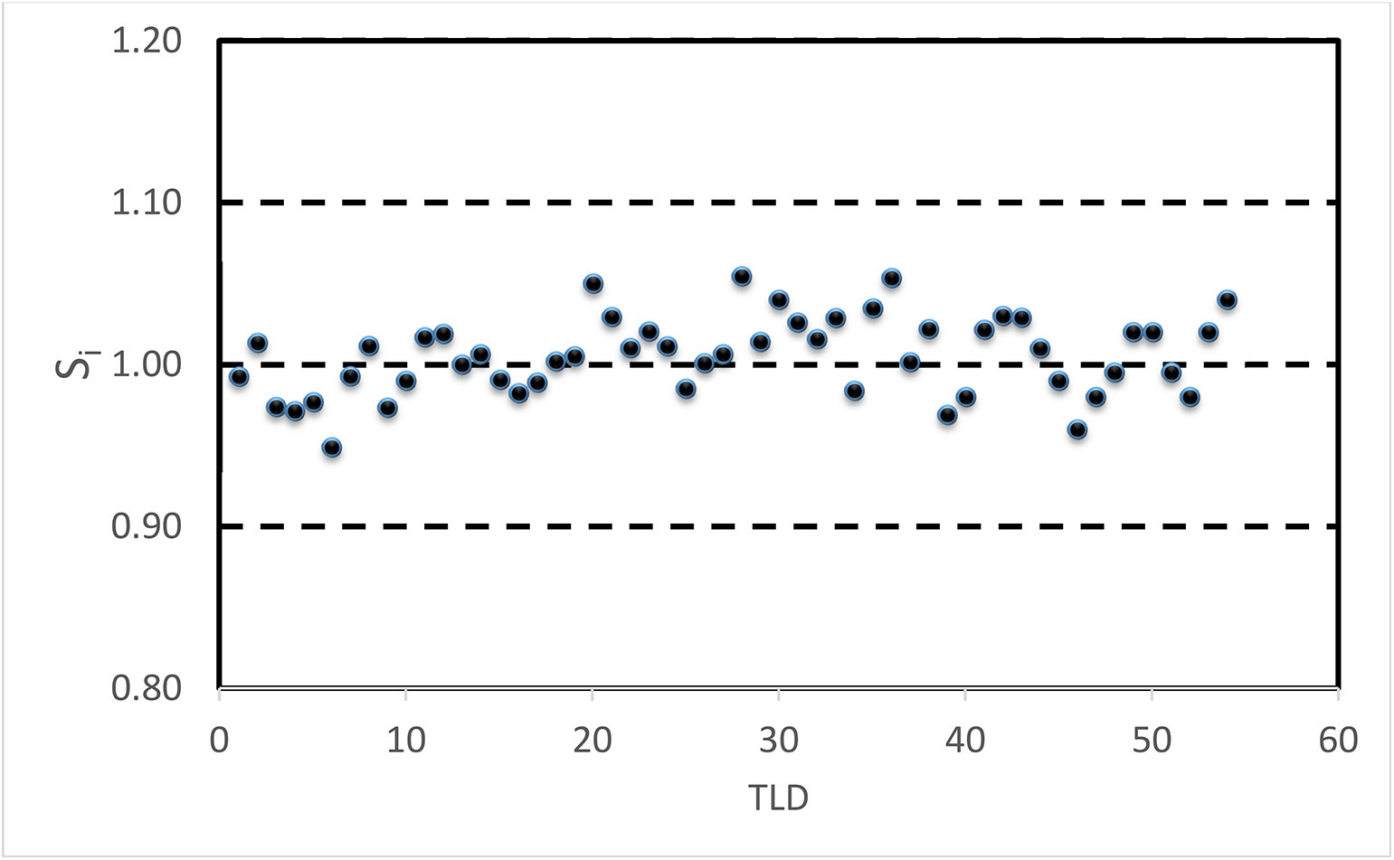
Individual sensitivity correction factor for all forty Thermoluminescent Dosimeters (TLDs).

Sensitivity correction factors for all TLDs were reported in Fig 3. The *S*_*i*_ factors ranged between 0.95 and 1.05. All values were included in the range of ± 10% of the mean value, and none of TLDs was rejected. Measurements from single TLD were corrected for the corresponding *Si* factor.

**Fig 4:**
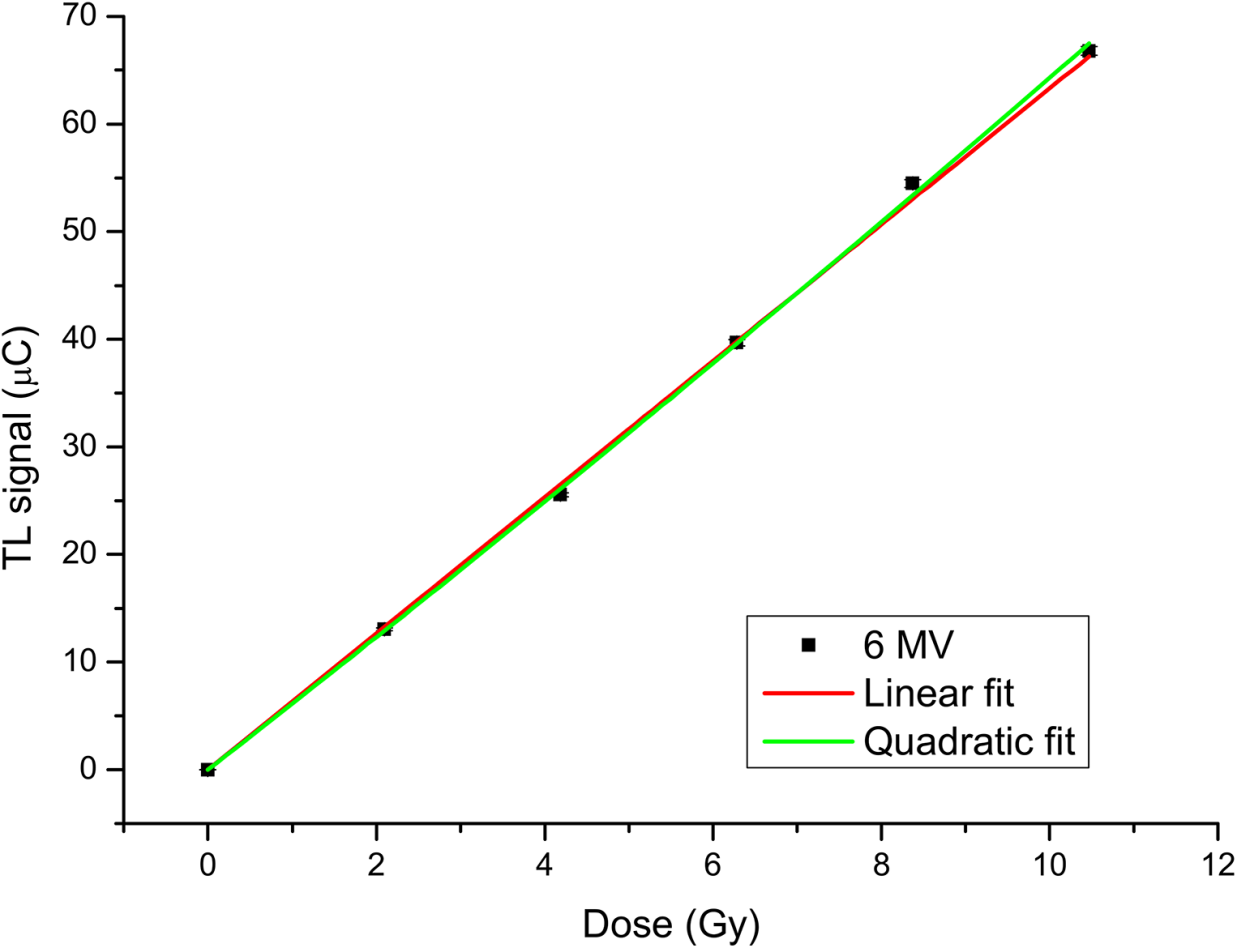
Thermoluminescent Dosimeters (TLDs) dose–response curve for doses between 0 Gy e 10 Gy at 6 MV photon beam.

The dose-response of TLDs for 6MV photon beam is depicted in Fig 4. The results from regression analysis show very high R^2^ values for both linear and quadratic models (respectively, R^2^ = 0.9995 and R^2^ = 0.9997). Since the two models are nested, the F-test approved the linear model as the best model (p = 0.169).

**Fig 5:**
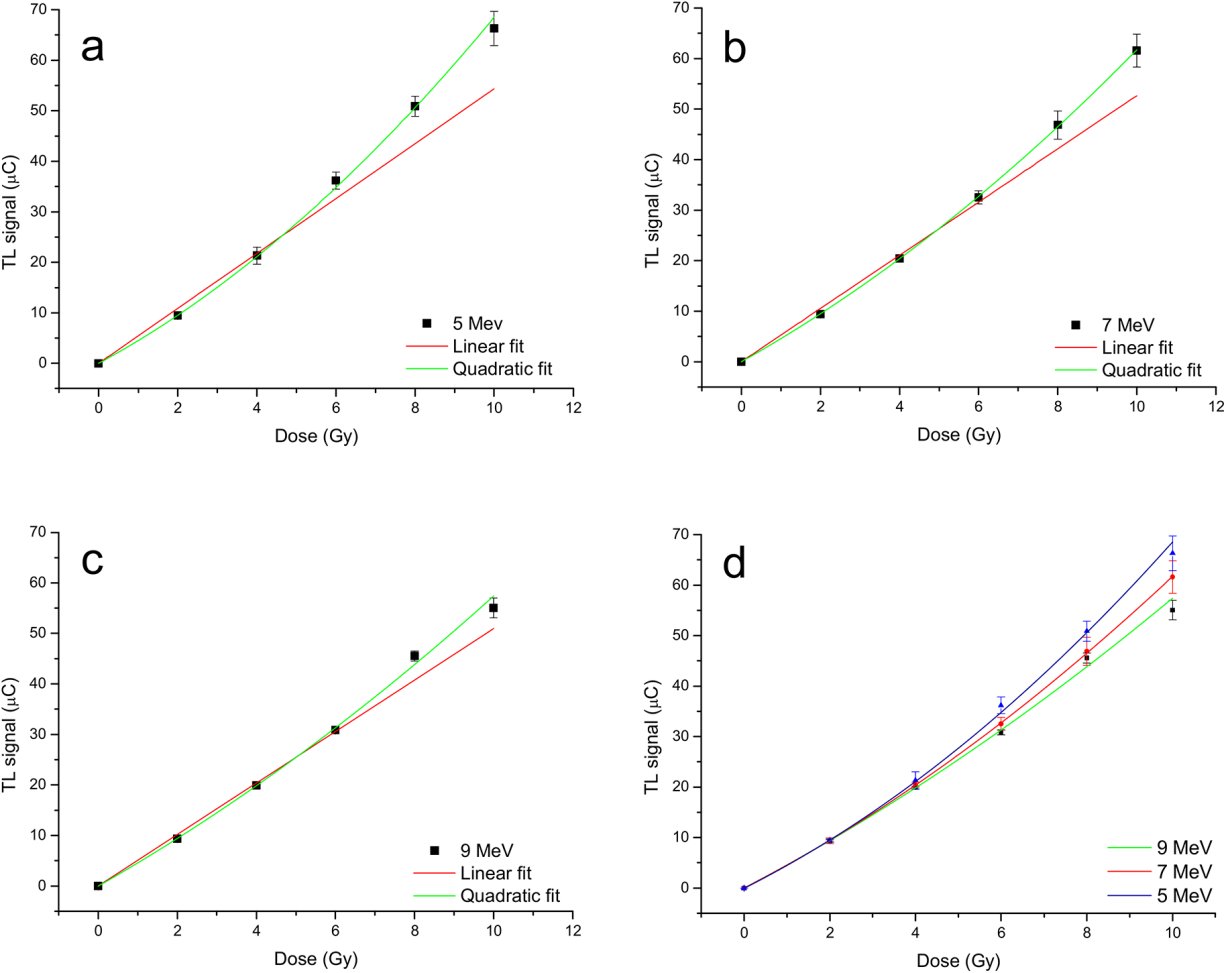
Thermoluminescent Dosimeters (TLDs) dose–response curve for doses between 0 Gy e 10 Gy. a) 5 MeV electron beam, b) 7 MeV electron beam, and c) 9 MeV electron beam. The red lines represent the linear fit, the green lines represent the quadratic fit. In d) the comparison between all curves is reported.

The TLD dose-response at 5 MeV electrons is depicted in Fig 5B. The linear model has an R^2^ = 0.974 while the quadratic model has an R^2^ = 0.999. For the 7 MeV and 9 MeV electron beams (Fig 5C and 5D), the R^2^ coefficients were 0.994 and 0.996 for the linear models, and 1.0 and 0.999 for the quadratic models, respectively. For all electron beams, the result of the F test approved the quadratic model (p<0.0004, Table 2). The best-fit regression coefficients for photon and electron beams were reported in Table 2.

**Table 2:** Photon and electron beams best-fit regression coefficients for the dose-response models.

Model	Linear				Quadratic				
Equation	y = A + B_1_ D				y = A + B_1_ D + B_2_ D^2^				*p*
		Coefficient	SE	Adj. R^2^		Coefficient	SE	Adj. R^2^	
**6 MV**	**A**	9.77E-4	9.93E-6	0.9995	**A**	9.77E-4	9.93E-6	0.9996	0.169
** **	**B**_**1**_ **(Gy** ^**-1**^ **)**	6.33	0.02		**B**_**1**_ **(Gy** ^**-1**^ **)**	6.09	0.05		
** **	** **				**B**_**2**_ **(Gy** ^**-2**^ **)**	0.03	0.01		
**5 MeV**	**A**	0.002	2.39E-04	0.9744	**A**	0.002	2.39E-4	0.9992	0.0014
	**B**_**1**_ **(Gy** ^**-1**^ **)**	5.43	0.11		**B**_**1**_ **(Gy** ^**-1**^ **)**	4.23	0.19		
					**B**_**2**_ **(Gy** ^**-2**^ **)**	0.26	0.03		
**7 MeV**	**A**	0.002	2.39E-04	0.9937	**A**	0.002	2.39E-4	0.9999	0.0002
** **	**B**_**1**_ **(Gy** ^**-1**^ **)**	5.27	0.09		**B**_**1**_ **(Gy** ^**-1**^ **)**	4.38	0.24		
** **	** **				**B**_**2**_ **(Gy** ^**-2**^ **)**	0.18	0.05		
**9 MeV**	**A**	8.37E-4	1.33E-04	0.9962	**A**	8.37E-4	3.4E-06	0.9993	0.0021
	**B**_**1**_ **(Gy** ^**-1**^ **)**	5.09	0.04		**B**_**1**_ **(Gy** ^**-1**^ **)**	4.44	0.11		
					**B**_**2**_ **(Gy** ^**-2**^ **)**	0.13	0.02		

Abbreviations: SE = Standard Error; Adj = Adjusted; R = Correlation coefficient.

## Discussion

A worldwide experience generated in the last 20 years has shown intraoperative radiation therapy to be a feasible technique for many cancer treatements. IOERT treatments have been used for gastric, pancreas, breast cancers and soft tissue sarcoma [1–3]. The aim of the IOERT is to improve local control and disease-free survival after surgical resection, particularly in a situation of close or positive margins. IOERT can be combined with external beam radiation therapy, or used as a single radiation dose to provide the best combination of loco-regional treatment [4]. The doses employed in IOERT ranged from 10 to 30 Gy [1–3] for exclusive IOERT treatment or 8–20 Gy if IOERT is used as additional boost [5, 6, 8–10, 33]. The administering of so high a level of radiation dose in a single fraction makes *in vivo* dosimetry for quality assurance of the IOERT mandatory.

MOSFET or microMOSFET dosimeters have been proposed for online in vivo dosimetry in IOERT procedures for different tumor types [13–15]. Gafchromic and radiochromic films wrapped in sterile envelopes for offline in vivo breast or rectal dosimetry [34, 35] were also used. Recently, Rabatjazi et al. [36] suggested the use of EBT2, a second generation of Gafchromic EBT film, for breast IOERT dosimetry verification.

MOSFETs have the advantage to provide an immediate reading, but the cables connecting the detectors to the reader might limit their usage. Indeed, the simultaneous insertion of more than one MOSFET in the surgical field can be demanding. On the other hand, the main drawback of Gafchromic films is the difficulty to shape them in small size since the cut edges of films should be avoided for dosimetry analysis [37].

Thermoluminescence dosimeters are routinely used in standard external beam radiotherapy to verify absorbed dose calculations at specific sites in a radiation field, either directly on a patient or in a phantom [19]. Their employment in IOERT has not been completely investigated yet. Fogg et al. described the only application of TLD for in vivo intraoperative radiotherapy [38]. The authors report on the use of TLDs for skin dosimetry during breast cancer treatments by 50 kV X-ray needle dosimetry.

Like radiochromic films, TLDs are cable free and do not provide online dose measurements. However, their size is smaller (few mm^3^) and offers the possibility of positioning more than one dosimeter simultaneously in the tumor bed in order to reconstruct tumor dose distribution. In addition, a TLD is reusable after annealing and can be easily sealed in a sterile envelope. All these characteristics make their use attractive for IOERT dosimetry in addition to an on-line dosimeter, i.e. MOSFET.

The thermoluminescence response curve shows a marked dependence on beam energy, radiation quality, modality and absorbed dose. When TLDs are irradiated by a conventional clinic linear accelerator (6–15 MV), the response curve is thought to be linear up to approximatively a few Gray. At higher doses, above 10 Gy, it exhibits a non-desirable non-linear behavior [24]. To our best knowledge, no data are present in the literature about the TLD dose-response when irradiated by IOERT beams.

In this framework, we have investigated the dose-response of commonly used commercial TLD dosimeter, LiF:Mg,Ti TLD-100. These dosimeters were irradiated with different high dose-per-pulse electron beams produced by an IOERT dedicated accelerator. The experiments were performed with different beam energies (5, 7 and 9 MeV) in the dose range of 0–10 Gy.

We first tested the accuracy of the whole experimental procedure with a conventional 6 MV photon beam, used as reference, then confirmed the expected linear behavior of TLD-100 for doses ≤10 Gy (Fig 4).

The response of TLDs, irradiated by high dose-per-pulse rate electron beams (2.6–3.1 cGy/pulse), could be still described by a linear function. However, we found that a quadratic function can better represent the response variation with the dose (Table 2). Indeed, the difference in R^2^ of the two model fits was small but significant (p<0.0004). Of note, the parabolic trend is more marked as the electron energy decreases (Fig 5). The second order coefficients for the 5 MeV and 9 MeV beams were 0.26 Gy^-2^ and 0.13 Gy^-2^ respectively. These results imply that an improvement in TLD calibration accuracy in high dose-per-pulse electron beams can be obtained if a quadratic dose-response correction is included.

The supralinear trend of TLD dose-response curve above a certain dose point has been explained with the development of additional lattice defects produced by irradiation in thermoluminescent crystals. These defects may act as electron traps and thus take part in the TL process causing an increase in detector response [39]. In our irradiation experiments, we observed a non-linear behavior for the dose-response functions already at lower dose-levels than those tipically reported for conventional photon beams. Futhermore, we observed that the variability of TL signal increses as the IOERT absorbed dose increases. We can speculate that these effects might be related to the high density of electric charge per pulse produced in the TL crystals by the used high dose-per-pulse beams or simply related to a larger damage creation by the electron beams.

In conclusion, we demonstrated the feasibility of *in vivo* dosimetry with TLD-100 in high dose-per-pulse beams to perform independent verification of treatment dose delivery. The non-linearity in the dose-response curve of TLD does not prevent their use for IOERT *in vivo* dosimetry, but it requires careful application of correction factors. Further studies are necessary to implement TLD employment in the clinical routine of IOERT.
